# Colony politics to chemical warfare: phenotypic plasticity in *Streptomyces*

**DOI:** 10.1042/EBC20250034

**Published:** 2026-09-02

**Authors:** John T. Munnoch, Paul A. Hoskisson

**Affiliations:** Strathclyde Institute of Pharmacy and Biomedical Sciences, University of Strathclyde, 161 Cathedral Street, Glasgow G4 0RE, U.K.

**Keywords:** antibiotics, development, natural products, plasticity, sporulation, Streptomyces

## Abstract

Members of the genus *Streptomyces* are prolific producers of antibiotics and bioactive natural products, whose biosynthesis is intricately linked to complex social and ecological interactions. Understanding how these bacteria adaptively regulate development and natural product biosynthesis in response to diverse environmental cues remains incomplete. Here, recent advances are synthesised revealing that phenotypic plasticity in *Streptomyces* is underpinned by regulatory networks integrating physiological and social signals, enabling multicellular differentiation, division of labour, and alternative growth modes such as exploration and foraging. These dynamic responses allow colonies to balance competition, cooperation, and reproduction in fluctuating environments. Our insights highlight the ecological and evolutionary significance of plasticity in shaping *Streptomyces* biology, with implications for natural product discovery and understanding microbial community dynamics.

## Introduction

Members of the bacterial genus *Streptomyces* are renowned as prolific producers of antibiotics and other bioactive natural products [1–3], and these compounds are increasingly recognised as the products of complex social and ecological interactions rather than isolated metabolic traits [3–6]. *Streptomyces* grow as multicellular, filamentous mycelia that can undergo coordinated developmental cycles resulting in differentiated hyphae and the terminal formation of spores, and this morphological differentiation is intimately linked with natural product biosynthesis [7]. The majority of *Streptomyces* species inhabit soil environments, existing within dense, competitive microbial communities, where survival depends on the ability to sense and respond to neighbours and flexibly deploy chemical and developmental strategies [4,5,8–11].

Phenotypic plasticity refers to the capacity of a genotype to produce diverse phenotypes under varying environmental conditions. Different genotypes emerge within populations (clones) and may also possess different plastic responses to specific environmental cues. Thus, genetic variation and the evolutionary potential it provides can generate differences in plasticity among populations [12]. Natural product biosynthesis, including antibiotics in *Streptomyces*, is now widely accepted as a context-dependent social behaviour [3–6,9,13,14]. At ecologically relevant concentrations, many natural products, including antibiotics, act not only as weapons but also as signalling molecules that modulate gene expression, development, and community structure [15–17]. Consistent with this view, the majority of biosynthetic gene clusters (BGCs) are conditionally expressed, responding to nutrient limitation, stress, population density, and intra- and interspecies interactions rather than being constitutively active [2,6,18–21]. Attempts to mimic ecological interactions through co-culture experiments and environmentally informed growth conditions have achieved initiation of developmental processes and activation of otherwise silent BGCs [14,22–29], serving to highlight that there is extensive phenotypic plasticity present in *Streptomyces*.

The phenotypic plasticity observed in *Streptomyces* is underpinned by regulatory networks that couple development, metabolism, and environmental sensing [5,6,20,21]. Global regulators such as BldD and AdpA, and a wide range of regulatory and signalling systems integrate nutritional, physiological, and social cues to coordinate antibiotic production with distinct types of developmental transitions, including sporulation, exploration, and foraging [9–11,13,29–37]. Importantly, the genetic architectures and mechanisms that facilitate phenotypic plasticity enable *Streptomyces* populations to balance competition, cooperation, and reproduction in dynamic environments.

In the present review, recent discoveries in the biology of *Streptomyces* are synthesised and reframed to discuss the ecological and evolutionary importance of plasticity in these organisms. Illuminating phenotypic plasticity in *Streptomyces* exemplifies the heterogeneity observed in the growth of these organisms, their response to environmental changes, and how this shapes their biology in complex and dynamic environments.

## Multicellularity and division of labour

*Streptomyces* are soil-dwelling Gram-positive bacteria that belong to the phylum Actinomycetota. These bacteria combine filamentous multicellular growth, extensive secondary metabolism, and colony-level organisation in a manner that, while mechanistically bacterial, possesses parallel features of multicellular eukaryotes [30,38]. These traits underpin their ecological success and the remarkable diversity of bioactive natural products produced by these organisms [2]. Increasingly we are recognising a role for phenotypic plasticity, colony heterogeneity, and division of labour traits to underpin *Streptomyces* biology within their spatially structured colonies.

**Figure 1:**
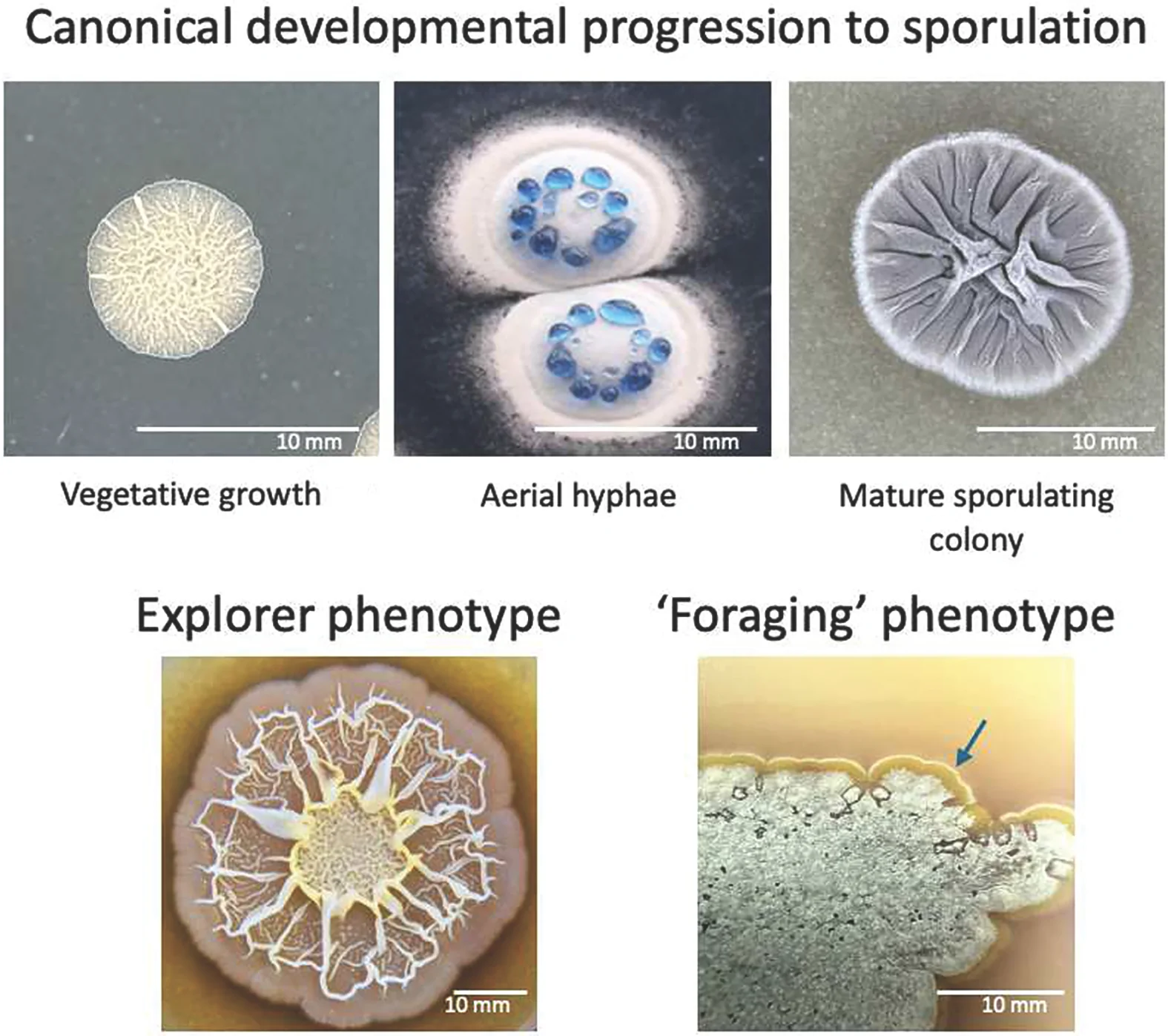
Phenotypic plasticity in *Streptomyces* development Canonical developmental progression of *Streptomyces* shows the transition from a vegetative colony (bald [*bld*]; no aerial hyphae) through the formation of aerial hyphae (white; *whi*) stage of development, often associated with the initiation of natural product production (in the case of the image, blue droplets of the polyketide actinorhodin can be seen) to the grey powdery appearance of the mature, sporulating colony. The description of the explorer phenotype shows large colonies formed of unbranched mycelium [28] that form in response to volatile signalling and exhaustion of specific carbon sources in the medium. Recently, the ‘foraging’ phenotype was described, which is characterised by the emergence of thin ‘veil’-like zones of growth at the edges of colonies that are distinct from the main colony [37]. The mechanistic detail that underpins this phenotype is still to be elucidated. All photographs by Prof. Paul A. Hoskisson.

The canonical *Streptomyces* life cycle (Figure 1) begins where a unigenomic spore germinates, growing apically from the tip, resulting in the formation of a branching filamentous mycelium that extends through the substrate [30,39]. This branched mycelium forms cross-walls (septa) infrequently, where growth can result in the formation of highly structured multicellular colonies comprised of physiologically distinct hyphae [30,40–42]. The hyphal network comprising the colony may experience a range of spatially and physiochemically heterogeneous external microenvironments, where variation in the nutrients, pH, oxygen, moisture, etc. must be integrated into cellular responses [6,43]. This integrated behaviour underpins key lifecycle decisions such as morphological and physiological differentiation.

In recent years we have come to appreciate that *Streptomyces* multicellularity extends beyond simple cycles of hyphal growth that encounter nutrient starvation and then initiate sporulation. There is increasing recognition that colonies compartmentalise activities such that different parts of the mycelium experience distinct developmental fates simultaneously [4,9,10,13], and this behaviour has features conceptually analogous to tissue specialisation in multicellular eukaryotes. The notion that *Streptomyces* should be considered as multicellular organisms with orchestrated cell behaviours rather than mere aggregates of independent cells is now widely accepted as part of their developmental biology.

A hallmark of social systems is the emergence of division of labour, where distinct subpopulations specialise in roles that enhance collective success [44]. Recent work on *Streptomyces* has provided compelling evidence for genomic heterogeneity leading to division of labour due to chromosome deletions, giving rise to cells that differ in gene content, such that hyper-producers of antibiotics emerge within colonies [10,45]. These antibiotic hyper-producing strains exhibit reduced individual fitness but enhance the overall fitness of the colony, while other clones within the colony contribute to spore formation [45]. Traditionally we have thought of *Streptomyces* as vegetative mycelium and reproductive structures (aerial hyphae and spores). The elegant work of Rozen and co-workers suggests that additional, asporulent cell types emerge through division of labour are analogous to worker castes in eusocial insects [45].

These observations closely match theoretical predictions about division of labour in cooperative organisms: specialised cells perform costly tasks that benefit the collective, even at an individual fitness cost [4,44]. Zhang et al. [45] showed that when mutant hyper-producer cells are mixed with wild-type cells, the overall colony produces more distinct natural products and a greater quantity of antibiotics without compromising overall spore production—essentially maximising colony fitness. These dynamics illustrate how *Streptomyces* implement a division of labour that balances reproduction and chemical defence [4,6]. The ecological drivers of division of labour may also extend to interactions with other organisms [3,5]. Interspecies competition can induce localised production of secondary metabolites and trigger the formation of specialised cell states within colonies [9,13]. Inhibition of growth by other *Streptomyces* is generally increased if strains are closely related, especially if BGCs are shared. Yet, remarkably, antibiotic production is less likely to be induced by growth-inhibiting competitor strains, suggesting that cellular damage is not a cue for inducing BGC expression [9]. These specific responses induced through interactions with other organisms further emphasise the social context of division of labour in *Streptomyces* and the range and complexity of behaviours these organisms are capable of.

## To sporulate or not, that is the question

Sporulation in *Streptomyces* represents a major developmental commitment that results in the formation of specific reproductive structures (aerial hyphae) that terminally differentiate to produce spores (Figure 1). Under standard laboratory conditions, this process follows a highly regulated process, where vegetative mycelium gives rise to aerial hyphae that undergo sporulation septation and maturation into unigenomic spores [6,30]. Many species of *Streptomyces* do not sporulate in submerged culture, but physiological differentiation, that is the production of natural products, still occurs [46–48]. One strain that has attracted much attention as a model due to complete and synchronous sporulation in liquid culture is *Streptomyces venezuelae* [49,50]. This has provided an excellent model for studying *Streptomyces* sporulation and cell biology [30]. While spores are produced in submerged and solid culture, it is worth noting that there are key differences in the spores produced under each condition. Spores from submerged cultures are formed without classical, hydrophobic aerial hyphae; spores are larger in size and have thinner walls compared with those formed on agar plates [51]. There are also key differences in the cell biology associated with the two spore types where localisation of proteins such as the actin homologue MreB is altered between the two conditions [51].

All known regulatory mutations that block sporulation in solid culture also block sporulation in submerged cultures of *S. venezuelae* [30]. Genes associated with aerial hyphae formation and regulation, cross wall formation, and even the formation of the hydrophobic sheath of aerial hyphae (used to break the surface tension during solid growth) are expressed during development in submerged culture [41,52–54]. These data serve to highlight submerged sporulation in *S. venezuelae* as an example of phenotypic plasticity, representing a rewired deployment of the canonical developmental programme under different physical constraints. These distinct phenotypic differences in *S. venezuelae* represent a clear opportunity and an excellent model to explore phenotypic plasticity in *Streptomyces* development.

Rather than being a uniform process across the entire colony in solid-grown cultures, sporulation often exhibits spatially stratified timing in *Streptomyces* [4–6]. Regions of the vegetative mycelium closer to resource exhaustion or competitive stress may initiate developmental processes earlier than in other regions [13]. This plasticity in timing ensures that the colony can hedge its bets, maintaining growth where possible while committing parts of the population to spore formation. Such a distributed responsive architecture is possible only in a multicellular system with extensive regulatory and communication networks.

Decades of research have focused on elucidating the mechanisms that underpin the developmental processes of *Streptomyces* (detailed summaries of this work can be found in [7,50,55,56]. The outcomes demonstrate that sporulation in *Streptomyces* relies on the interplay of multiple genetic and physiological inputs and regulatory mechanisms, rather than a single linear pathway. These regulatory networks integrate internal developmental signals, environmental cues, and molecular checkpoints to decide when and where sporulation will occur.

The master regulator of sporulation in *Streptomyces*, BldD, has its activity controlled by levels of the second messenger, c-di-GMP [32,57,58]. Experimentally modulating levels of c-di-GMP in cells alters BldD activity [57]. BldD is a c-di-GMP-binding regulatory protein, and the ability of BldD to bind to DNA target sequences requires c-di-GMP interaction [57]. BldD acts as a master repressor of sporulation, such that when in complex with c-di-GMP and bound to its DNA targets, it blocks entry into sporulation and prolongs vegetative growth [32,33,57,59]. Overexpression of diguanylate cyclases (DGCs; synthesising c-di-GMP) results in a conventional *bld* phenotype where the initiation of sporulation is blocked, whereas over-expression of specific phosphodiesterases (PDEs; degrading c-di-GMP) results in precocious hypersporulation [32]. *Streptomyces* species possess multiple genes associated with synthesis and/or degradation of c-di-GMP, and often these proteins contain multiple regulatory/sensing domains (e.g. GAF, PAS, and PAC) to modulate input signals [32]. While we currently do not know what regulatory inputs all of these DGC/PDEs respond to, at least four of the DGCs are direct targets of BldD, suggesting there is some negative regulation of c-di-GMP levels occurring once the onset of sporulation occurs [58], although interplay between all of the DGC/PDEs still remains to be elucidated. Moreover, these data suggest that input signals to initiate development are multiple, complex, and parallel in terms of regulating the onset of sporulation in *Streptomyces*, exemplifying extensive plasticity in this process.

**Figure 2:**
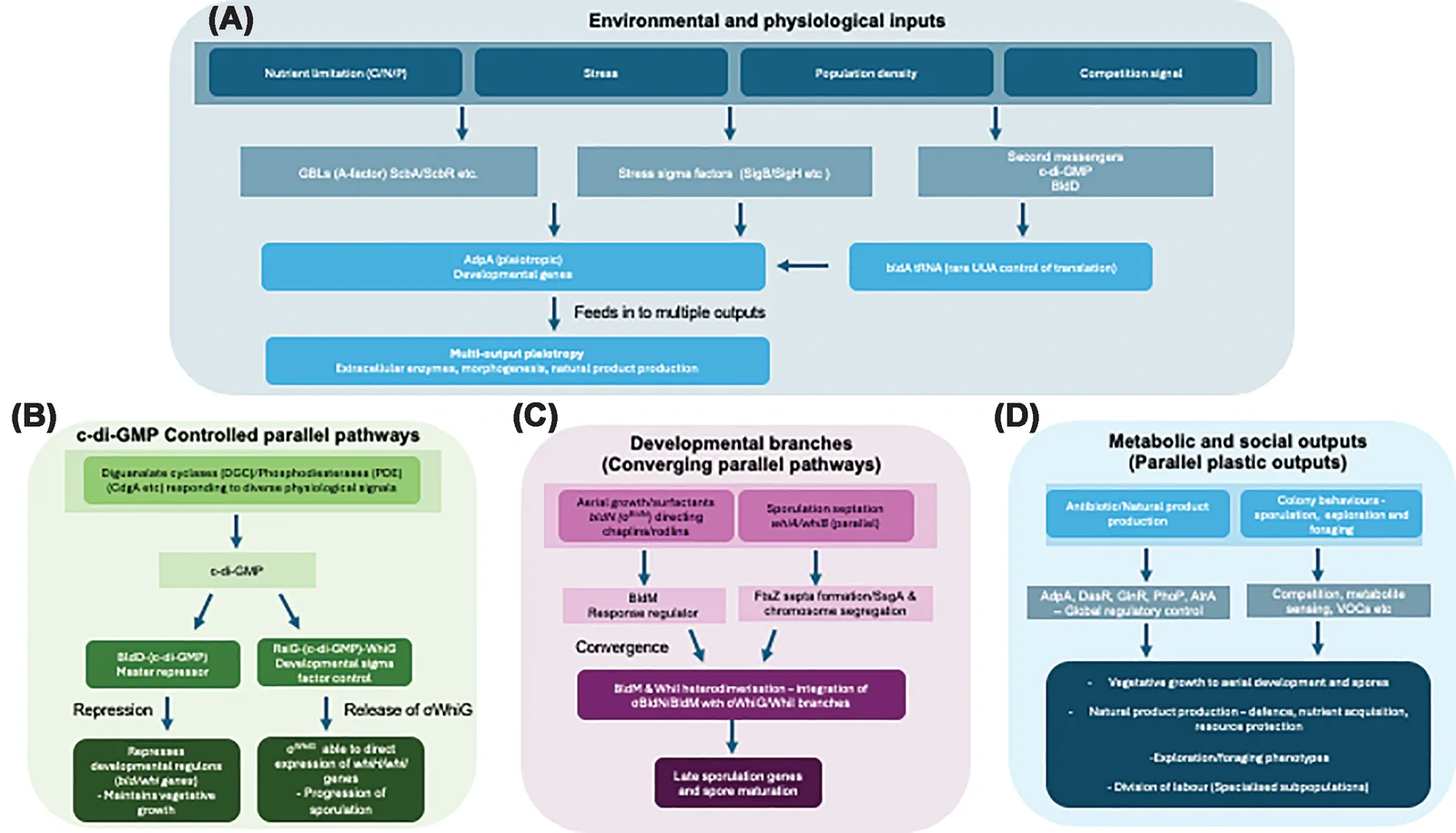
Parallel and convergent regulatory routes leading to plasticity *Streptomyces* development (**A**) Environmental and physiological inputs (nutrient limitation, stress, and competition) are integrated through several regulatory modules that operate partly in parallel. γ-Butyrolactone (GBL) signalling regulates pleiotropic transcription factors such as AdpA. (**B**) While global physiological control is mediated by c-di-GMP signalling, which modulates both BldD-dependent repression and σ^WhiG^ activity via RsiG. (**C**) Distinct developmental branches (aerial development via σ^BldN^ and sporulation via σ^WhiG^ and other *whi* genes) converge at key integration points, including BldM–WhiI heterodimer formation. (**D**) These branched architectures facilitate plasticity in development and couple morphogenesis to natural product biosynthesis. See text for specific details.

This is not the only point in the development of *Streptomyces* where c-di-GMP acts to modulate progression through sporulation. Notably, c-di-GMP also controls sequestration of the sporulation sigma factor σ^WhiG^ via the anti-sigma factor RsiG [32,60]. RsiG interacts with c-di-GMP, such that when levels fall, σ^WhiG^ is released from RsiG to activate late sporulation genes [32,60]. These two regulatory proteins (BldD and σ^WhiG^) that bind c-di-GMP represent parallel regulatory branches that couple physiological state to distinct developmental regulators in the network (Figure 2). Extensive studies of the regulatory architecture that governs *Streptomyces* sporulation have revealed that the overall nature is modular and network-like rather than linear. While BldD is the master regulator of sporulation [32], binding directly to the promoters and repressing a wide regulon of developmental genes including during vegetative growth, including signal transduction components, sigma factors, anti-sigma factors, and key regulators [58]. The targets of BldD include *bldA, bldC, bldM, bldN, ssgA/B, ftsZ, whiB*, and *whiG*, indicating that BldD-mediated repression encompasses both aerial growth and spore formation/maturation [58]. Once c-di-GMP levels fall and BldD repression of development is relieved, multiple downstream pathways are derepressed simultaneously, not in a simple one-after-the-other type cascade of gene expression. Further highlighting the plastic nature of development in *Streptomyces* are two further, independently regulated, converging pathways in sporulation [7]. The *whiA* and *whiB* genes are regulated independently of the sigma factor *whiG*, which, in turn, controls the *whiH* and *whiI* regulatory genes. These branches within sporulation are distinct yet converging pathways that are required for proper sporulation [7]. Furthermore, while *whiB* is a target for repression via BldD, there is a further, dedicated regulator of *whiB* called BldO [61]. It is clear that in early development BldO represses *whiB*; however, when repression is relieved, WhiA and WhiB somehow combine to stimulate expression of *bldO* [61], which results in the repression of *whiB*. This suggests that *whiB* expression is transient and tightly controlled. Intriguingly, BldO is a MerR-like regulator that possesses a ligand-binding domain, suggesting that further, as yet unknown physiological signals are integrated into sporulation via BldO.

Perhaps the most striking plastic response in *Streptomyces* development is the role played by the atypical response regulators BldM and WhiI [62], which are able to integrate parallel developmental signals (Figure 2). BldM is a target of the master regulator of development, BldD [58]. BldM can act as a homodimer and is responsible for activating a set of early sporulation genes (Group 1 genes) [63]. WhiI is required later in development for the formation of sporulation septa and chromosomal condensation [64], and its activity is dependent on the sporulation sigma factor WhiG [7]. Remarkably, the formation of a BldM/WhiI heterodimer is able to activate a distinct set of developmental genes (Group 2 genes) later in development [63]. This heterodimer formation integrates signals from the σ^WhiG^ into the WhiI pathway and the σ^BldN^/BldM pathway, illustrating how two upstream branches converge via a combinatorial protein complex, providing a mechanism to expand signalling capability and provide further plasticity through BldM acting at two distinct points in *Streptomyces* development [63].

GBLs are small diffusible signalling molecules that act as quorum-like regulators in *Streptomyces* and appear to coordinate secondary metabolism and development in response to population density and the physiological state of cells [65]. The archetypal system is A-factor in *Streptomyces griseus*, which accumulates during growth and binds the TetR-family receptor ArpA, relieving repression of *adpA* (a pleiotropic regulatory gene) that activates sporulation and antibiotic production (notably streptomycin biosynthesis in *S. griseus*) [66,67]. Homologous GBL systems exist in *Streptomyces coelicolor*, including the wSCB system (ScbA/ScbR), regulating antibiotic biosynthetic pathways such as actinorhodin, undecylprodigiosin, and coelimycin with cross-talk between global and BGC-situated regulators along with indirectly affecting development [65]. GBL signalling is now understood as an additional layer of signalling and regulation that is integrated into decision-making in *Streptomyces*. Many *Streptomyces* species encode diverse GBLs and butyrolactone-like ligands (such as 2-alkyl-4-hydroxymethylfuran-3-carboxylic acids [68]; butenolides [69,70]), their receptors, and biosynthetic enzymes, which are often linked to BGCs and development, enabling fine-tuned activation of costly biosynthesis and development only under appropriate conditions [71].

We currently do not fully understand how GBLs integrate into the wider developmental processes of *Streptomyces.* GBL signalling provides a population-density (‘social’) cue that activates developmental and biosynthetic regulators, while c-di-GMP modulation of BldD and σ^WhiG^/RsiG imposes a physiological (private) checkpoint that ensures social signals are integrated with a metabolic readiness to initiate aerial hyphae formation and natural product production. This convergence on the same developmental checkpoint, which appears to be AdpA in the first instance, creates plasticity and allows for each to partially gate the effectiveness of the other (Figure 2).

## Exploratory and foraging phenotypes expanding developmental plasticity in *Streptomyces*

Beyond the classical sporulation lifecycle, *Streptomyces* can manifest alternative growth modes that exemplify behavioural and regulatory plasticity (Figure 1). A striking example is the exploratory growth phenotype, originally described in *Streptomyces venezuelae*, whereby colonies abandon typical filament branching and instead adopt a non-branching, rapid surface translocation mode (Figure 1) [29]. This behaviour does not rely on the classical developmental cues but utilises metabolic signals and inter-domain interactions, such as co-culture with fungi. Fungi can trigger this behaviour through altering the local growth environment in a manner that provokes *Streptomyces* to switch into an exploratory state [29]. Explorer cells are able to communicate this behaviour over longer distances via volatile organic compound signalling, which can induce exploration and modulate nutrient acquisition factors such as siderophores in *Streptomyces* colonies [29,31,72], promoting collective behaviours across spatially distinct colonies. The addition of certain carbon sources is also able to align the exploratory phenotypes with that of the conventional sporulation pathways [31]. Remarkably, mutations in the classical developmental genes did not affect exploration, but strains exhibiting precocious sporulation were unable to activate the exploratory phenotype [31]. Moreover, beyond the developmental aspects of exploration phenotypes in *Streptomyces*, exploration also results in alterations of cell wall biosynthesis and how it is directed in explorer cells, resulting in structurally distinct cell walls compared with that of conventional *Streptomyces* hyphae [39].

Recently, a previously unidentified example of phenotypic plasticity has been described, termed ‘foraging’, due to continuous growth on exhausted media at the edge of a colony (Figure 1) [37]. The authors suggest that the ‘foraging phenotype’ is different from conventional colony phenotypes in terms of morphology, the colonies exhibit none of the genomic instability associated with division of labour and the metabolomic profile is distinct, including the activation of antimicrobial compound production [37]. The ‘foraging phenotype’ has been proposed as an adaptation to nutrient deprivation and appears to be reproducible across the *Streptomyces* genus. While this work raises interesting questions and adds to the repertoire of plastic phenotypic responses of *Streptomyces*, more work is required to fully elucidate the molecular mechanisms that underpin it.

Exploration [29] (and potentially the ‘foraging phenotype’ [37]) appears to function as a foraging strategy (spatial expansion and changes to nutrient acquisition behaviour), enabling colonies to rapidly colonise new territory in response to competitive or nutritional cues. In the heterogeneous and dynamic environments that *Streptomyces* inhabit, such phenotypic plasticity is likely adaptive and enhances ecological success.

## Phenotypic plasticity as a feature of natural product biosynthesis in *Streptomyces*

Biosynthesis of natural products in *Streptomyces* is a highly regulated and environmentally contingent trait. A major driver of phenotypic plasticity in this context is the integration of extracellular signals with social and physiological regulatory networks that enable cells to balance growth, development, and production of bioactive natural products [73]. Beyond canonical GBL systems that regulate BGC activation, natural products and their biosynthetic intermediates frequently function as signalling molecules, feeding back to their own biosynthetic clusters or cross-regulating other pathways [9,21,28,74–85]. These scenarios create dense regulatory topologies capable of context-dependent switching between alternative chemical repertoires in strains in response to stimuli. This regulatory and phenotypic plasticity likely reflects the ecological role of antibiotics not only as agents of chemical warfare but also as signalling or niche-altering molecules that can be deployed strategically in complex communities.

## Integrative perspective

The social life of *Streptomyces* emerges from the intersection of multicellularity, phenotypic plasticity, and division of labour. Increasing evidence suggests that we should move away from the view that *Streptomyces* colonies are not mere aggregates of clones but dynamic, spatially structured entities capable of generating heterogeneous and plastic phenotypic responses across multiple ecological challenges. Developmental programmes such as sporulation are contextual rather than deterministic, dynamically responding to cell physiology (private signals) and external signals and cues (social and ecological signals). Alternative development such as the exploration phenotype demonstrates that the classical view of the *Streptomyces* life cycle is incomplete without recognising the behavioural flexibility afforded by ecological interactions and dynamics. Emerging phenotypic traits such as foraging also indicate that for many years we have overlooked phenotypic plasticity in these organisms. *Streptomyces* colonies integrate signals and cues spatially and temporally to initiate complex morphological and physiological developmental processes. Understanding these processes at the mechanistic and ecological level promises to deliver insights into *Streptomyces* biology and also reveal strategies for activating silent BGCs and manipulating microbial communities.

## Summary

- Natural product biosynthesis and morphological development in *Streptomyces* are the product of complex social and ecological interactions rather than isolated metabolic traits.
- Survival in the natural environment depends on the ability to sense and respond to the environment and neighbouring species and flexibly deploy chemical and developmental strategies.
- Phenotypic plasticity, that is, the capacity of a genotype to produce diverse phenotypes under varying environmental conditions, in *Streptomyces* is underpinned by regulatory networks that couple development, metabolism, and environmental sensing.
- Environmental and population-density (‘social’) cues in *Streptomyces* integrate with physiological (‘private’) checkpoints to determine metabolic readiness to initiate development.
- Beyond canonical quorum-sensing-like systems, natural products and their biosynthetic intermediates frequently function as signalling molecules via regulatory feedback loops to their own biosynthetic clusters or cross-regulation of other pathways.

## Open Access

Open access for this article was enabled by the participation of the University of Strathclyde in an all-inclusive Read & Publish agreement with Portland Press and the Biochemical Society under an agreement with JISC.

## Abbreviations

BGCs: biosynthetic gene clusters
DGCs: diguanylate cyclases
GBLs: γ-butyrolactones
PDEs: phosphodiesterases
